# The Free T3/Free T4 Ratio Predicts Relapse After Antithyroid Drug Withdrawal in Pediatric Autoimmune Hyperthyroidism

**DOI:** 10.1210/clinem/dgag130

**Published:** 2026-03-31

**Authors:** Marco Abbate, Gaia Vincenzi, Stefano Mora, Giulia Tarantola, Ilenia Teresa Petralia, Cristina Santagiuliana, Luisa Del Giacco, Sara Zanelli, Maria Cristina Vigone

**Affiliations:** Department of Pediatrics, Endocrine Unit, IRCCS San Raffaele Scientific Institute, Milan 20132, Italy; Department of Pediatrics, Endocrine Unit, IRCCS San Raffaele Scientific Institute, Milan 20132, Italy; Laboratory of Pediatric Endocrinology, Department of Pediatrics, IRCCS Ospedale San Raffaele, Milan 20132, Italy; Department of Pediatrics, Endocrine Unit, IRCCS San Raffaele Scientific Institute, Milan 20132, Italy; Department of Pediatrics, Endocrine Unit, IRCCS San Raffaele Scientific Institute, Milan 20132, Italy; Department of Pediatrics, Endocrine Unit, IRCCS San Raffaele Scientific Institute, Milan 20132, Italy; Department of Pediatrics, Endocrine Unit, IRCCS San Raffaele Scientific Institute, Milan 20132, Italy; Department of Pediatrics, Endocrine Unit, IRCCS San Raffaele Scientific Institute, Milan 20132, Italy; Department of Pediatrics, Endocrine Unit, IRCCS San Raffaele Scientific Institute, Milan 20132, Italy; Department of Pediatrics, Endocrine Unit, Vita-Salute San Raffaele University, IRCCS San Raffaele Scientific Institute, Milan 20132, Italy

**Keywords:** autoimmune hyperthyroidism, relapse, FT3/FT4 ratio

## Abstract

**Context:**

The likelihood of remission in patients with autoimmune hyperthyroidism (AH) depends on several prognostic factors. The free T3 (FT3)/free T4 (FT4) ratio has been suggested as a potential marker of disease severity and prognosis.

**Objective:**

To evaluate the prognostic value of the FT3/FT4 ratio in predicting the likelihood of relapse in patients with AH.

**Methods:**

This retrospective cohort study included 80 patients with AH diagnosed between 2000 and 2021. Clinical and biochemical data were collected at diagnosis and during follow-up to assess the likelihood of remission in patients attempting to discontinue antithyroid drug (ATD) therapy. Receiver operating characteristic analysis and logistic regression were used to identify predictors of relapse.

**Results:**

Of the 48 patients who discontinued ATD therapy, 24 (50%) relapsed, predominantly within 12 months. The FT3/FT4 ratio was significantly higher in patients who relapsed both at diagnosis (median 0.46 vs 0.36; *P* .001) and at ATD withdrawal (median 0.43 vs 0.33; *P* < .001). An FT3/FT4 ratio ≥0.42 at diagnosis predicted relapse with 83% sensitivity and 66% specificity. In a multivariate analysis, FT3/FT4 ratio ≥0.42 at diagnosis (odds ratio 7.18; *P* .012) and the presence of orbitopathy were identified as independent predictors of relapse. Longer time to FT3 normalization (*P* .02) and orbitopathy were associated with relapse in univariate analyses (*P* .039).

**Conclusion:**

The FT3/FT4 ratio, measured both at diagnosis and before ATD withdrawal, is a significant predictor of relapse in AH. This parameter may represent a useful tool for guiding decisions about treatment duration and withdrawal.

Autoimmune hyperthyroidism (AH), predominantly caused by Graves' disease (GD), represents the most common cause of thyrotoxicosis in children and adolescents (1). The current first-line treatment for AH is antithyroid drugs (ATD), specifically methimazole (2, 3). However, remission after ATD withdrawal is achieved in only a minority of patients, with reported rates of approximately 20% to 30% after 2 years of therapy (4, 5). Consequently, the best approach for managing childhood-onset AH is still debated and varies across different countries. The American Thyroid Association recommends treatment with ATD for a period of 1 to 2 years, and definitive treatment is advised if no remission is achieved (2). The European Thyroid Association recommends a longer duration of ATD therapy of a minimum of 3 years and as long as 5 years or more in patients with a low likelihood of remission (3). A longer duration of treatment has been shown to be associated with higher remission rates (6-10).

Several clinical and biochemical factors have been identified as contributing to an enhanced probability of remission subsequent to ATD treatment. These factors include advanced age, female sex, Caucasian ethnicity, small goiter size, mild biochemical derangement at diagnosis, lower thyrotropin receptor autoantibody (TRAb) titer, a history of other autoimmune conditions, and duration of ATD treatment (5, 6, 8-14).

Among biochemical parameters, the relative pattern of thyroid hormone secretion may provide additional prognostic information beyond absolute hormone concentrations. In particular, the ratio between free T3 (FT3) and free T4 (FT4), which reflects preferential T3 production and intrathyroidal deiodinase activity, may capture disease characteristics associated with persistence and relapse (15, 16).

This biochemical phenotype, often referred to as T3-predominant hyperthyroidism, has been associated with more severe disease and poorer therapeutic response in adult populations (17, 18). Despite this biological rationale, the prognostic role of the FT3/FT4 ratio in predicting relapse after ATD withdrawal has been scarcely investigated in pediatric patients.

We therefore conducted a retrospective cohort study of children and adolescents with AH treated with methimazole at a tertiary pediatric endocrinology center. The primary objective was to assess whether the FT3/FT4 ratio at diagnosis and at ATD withdrawal predicts relapse after treatment discontinuation. Secondary objectives included the evaluation of other clinical and biochemical factors associated with disease outcome. This study seeks to provide clinically useful evidence for personalized management of pediatric AH by identifying easily accessible predictors of relapse.

## Materials and Methods

### Study Design and Population

The present retrospective study included 80 children and adolescents (aged under 18 years) diagnosed with AH and followed at the Paediatric Endocrinology Centre of the IRCCS San Raffaele Hospital (Milan, Italy) between 2000 and 2021. The diagnostic criteria for AH are as follows: the presence of clinical signs and symptoms of hyperthyroidism, suppressed TSH, elevated free thyroid hormones, positivity for thyroid antibodies, and ultrasound features consistent with autoimmune thyroid disease. The initial dose of methimazole administered to all patients ranged from 0.15 to 0.65 mg/kg/day. The therapeutic dosage was adjusted on the basis of serum TSH and free thyroid hormone values, with the association of L-thyroxine in selected cases using a block and replace approach (in the presence of TSH and FT4 values lower than normal limits and FT3 values higher than normal limits) (19).

Patients were excluded if they had subclinical hyperthyroidism, neonatal hyperthyroidism, a follow-up duration of less than 24 months, or incomplete clinical or biochemical data. The study was conducted in accordance with the Helsinki Declaration, and all data were anonymized. Ethics committee approval was not required, since the General Authorization to Process Personal Data for Scientific Research Purposes exempts retrospective archive studies that use identifier codes preventing subject reidentification (Guarantees for the Protection of Personal Data, 2014; Authorization No. 9/2014—General Authorization to Process Personal Data for Scientific Research Purposes (online). Docweb No. 3632879, Garante Privacy].

### Data Collection

Clinical data collected at diagnosis included age, sex, pubertal stage (Tanner stage), and body mass index SD score. Additionally, family history of autoimmune thyroid disease; associated autoimmune or genetic conditions; and the presence of clinical signs and symptoms, such as thyromegaly, orbitopathy, tachycardia (with reference to normal values for age), and weight loss were documented. The severity of the disease at the time of presentation was categorized according to the number of clinical manifestations (≤2 vs >2).

The biochemical variables included serum TSH, FT3, FT4, FT3/FT4 ratio, and thyroid autoantibodies (TRAb, thyroperoxidase antibodies, thyroglobulin antibodies). Thyroid ultrasounds and antithyroid antibody measurements were conducted approximately once a year. During the follow-up, we collected the following data: time to normalization of FT3 and FT4 after starting therapy, TRAb negativization and negativization time, duration of the first cycle of methimazole therapy, biochemical values before discontinuing therapy, value of TRAb before discontinuing therapy, dose of methimazole (mg/kg/day) before discontinuing therapy, duration of suspension and possible onset of relapse, and definitive therapy. Relapse was defined as suppressed TSH associated with elevated FT3 and FT4 concentrations after ATD discontinuation. Remission was defined as maintenance of euthyroidism for a period of at least 12 months following treatment withdrawal or the progression to hypothyroidism requiring levothyroxine replacement.

#### Laboratory assessments

The hormonal assays of thyroid function were performed on serum using commercial kits that employ an immunoassay method. The FT3 value was expressed in pmol/L, the FT4 value in pmol/L, the TSH value in mcU/mL, and the FT3/FT4 ratio in pmol/pmol. In cases where values exceeded the upper reporting limit, the maximum reportable value was recorded and used for analysis, as no further dilution-based quantification was systematically available in the archived data. The presence of autoantibodies was verified through the use of commercial kits that use an immunoassay method (TRAb catalog #08496609190, RRID:AB_3696448; thyroperoxidase catalog #07026935190, RRID:AB_ 3675830; thyroglobulin catalog #07026919190, RRID:AB_ 2894922). The value of the autoantibodies was expressed as a multiple of the upper normal limit. The following values were consequently identified: negative, low titer positive (<2 times the upper normal limit), medium-low titer positive (2-5 times the normal upper limit), medium-high positive titer (5-10 times the upper normal limit), and positive with high titer (>10 times the upper normal limit).

#### Thyroid ultrasound

Thyroid ultrasound examinations were performed during follow-up by experienced operators. In view of the incomplete availability of all thyroid dimensions, analysis was limited to the anteroposterior diameter of each thyroid lobe. This was compared with reference values to age and sex. In particular, we calculated and reported the difference between each patient's lobe measurement and the age- and sex-specific upper reference value derived from the study by Suzuki et al (20). If there was no difference or the patient's lobe was smaller than the specific upper reference value, the value reported was 0.

#### Statistical analysis

The results obtained are expressed as percentages for categorical variables and as medians (25th-75th percentiles) for continuous variables. To compare the groups, Fisher's exact test was employed for categorical variables and the Mann-Whitney test for continuous variables. Receiver operating characteristic curve analysis was utilized to identify the optimal cutoff values for continuous predictors.

Variables associated with relapse in univariate analysis were entered into multivariate logistic regression models to identify independent risk factors. Statistical analyses were performed utilizing GraphPad Prism 10.2.2. A 2-sided *P*-value of less than .05 was considered statistically significant.

## Results

The study population includes 80 subjects suffering from AH. Baseline demographic, clinical, and biochemical characteristics are summarized in Table 1. The cohort was predominantly female (female-to-male ratio 3.7:1), with a median age at diagnosis of 10.8 years. The majority of patients were Caucasian (84%), and 31% reported a first- or second-degree family history of autoimmune thyroid disease. Associated autoimmune diseases or genetic syndromes were present in 29% of patients (celiac disease, type 1 diabetes mellitus, arthritis rheumatoid, autoimmune hepatitis, autoimmune thrombocytopenia and neutropenia, autoimmune gastritis, Down's syndrome).

**Table 1:** Baseline characteristics of patients with autoimmune hyperthyroidism

	n	Median (25th-75th percentile) or %	N
Sex (F/M)	63/17	79/21	80
Age (years)		11 (9-14)	80
Ethnicity (Caucasian/not Caucasian)	67/13	84/16	80
Pubertal stage (Tanner stage)			80
T1	38	47	
T2-T4	18	23	
T5	24	30	
Family history of AITD	25	31	80
Other autoimmune diseases/syndromes	23	29	80
BMI **(SD)**		−0.88 (−1.84-0.02)	80
Thyreomegaly	74	92	80
Orbitopathy	23	29	79
Weight loss	51	64	80
Tachycardia	59	74	80
Clinical presentation			79
Severe	52	66	
Not severe	27	34	
TSH (mcU/mL)		0.0055 (0.005-0.01)	80
FT3 (pmol/L)		23.94 (18.23-32.51)	80
FT4 (pmol/L)		54.56 (39.89-70.14)	80
FT3/FT4		0.44 (0.36-0.51)	80
TRAb			80
Negative	2	2.5	
< 2×	2	2.5	
2-5×	15	19	
5-10×	23	29	
>10×	38	47	
TPOAb			80
Negative	15	19	
< 2×	5	6	
2-5×	17	21.5	
5-10×	10	13	
>10×	32	40.5	
TgAb			80
Negative	27	35	
<2×	13	17	
2-5×	8	10	
5-10×	9	12	
>10×	21	26	
LAPD (mm)		2 (0-4)	73
RAPD (mm)		0.5 (0-3)	73
First cycle of methimazole (years)		2.5 (2-4)	79
Time of follow-up (years)		5 (3-8)	80

Data are presented as median (interquartile range) or number (percentage).

Abbreviations: AITD, autoimmune thyroid disease; BMI, body mass index; FT3, free T3; FT4, free T4; LAPD, left anteroposterior diameter; RAPD, right anteroposterior diameter; TgAb, thyroglobulin antibodies; TPOAb, thyroperoxidase antibodies; TRAb, thyrotropin receptor autoantibodies.

**Figure 1:**
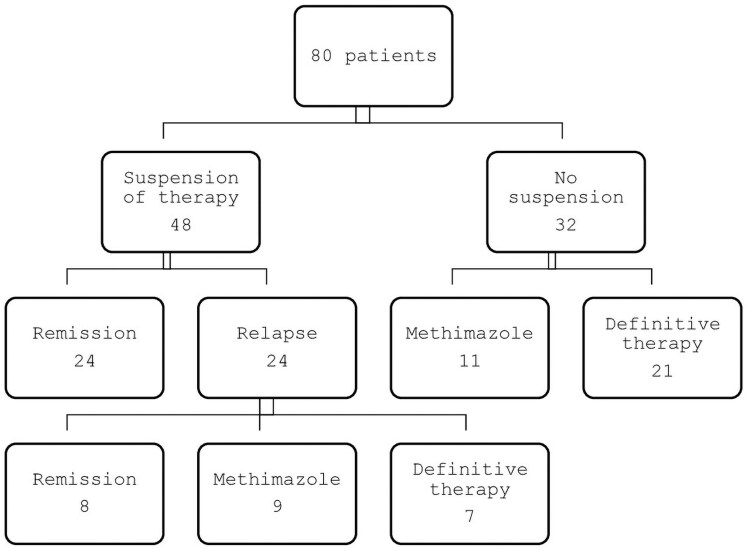
Flow diagram summarizing treatment course and final outcomes, including remission, relapse, ongoing antithyroid drug therapy, and definitive treatment (thyroidectomy or radioactive iodine). Median follow-up was 5 years. Forty-eight of 80 patients made at least 1 attempt to discontinue therapy. After the first cycle of therapy, 24 (50%) of them achieved remission, while the remaining 50% relapsed. After a second or third discontinuation attempt, another 8 patients (16.5%) achieved remission.

The mean follow-up was 6.1 years (median 5 years). The median duration of the first course of methimazole therapy was 3.2 years. During follow-up, 48 patients (61%) discontinued ATD therapy; of these, 24 (50%) experienced relapse and resumed treatment, whereas 24 (50%) achieved remission after the first withdrawal. Overall, 32 patients (40%) were in remission at last follow-up, 28 (35%) underwent definitive therapy, and 20 (25%) were receiving ongoing methimazole treatment (Fig. 1).

Table 2 shows the comparison between patients who relapsed and those who went into remission. Patients who suspended therapy took methimazole for an average duration of 2.7 years; we found a longer duration of the first cycle of therapy in the remission group although a significant difference was not observed. Relapse occurred within the first 12 months of suspension in 96% of patients. A family history of autoimmune thyroid disease and orbitopathy was significantly more frequent among patients who relapsed (45.8% vs 12.5%, *P* .024; 35% vs 8%, *P* .036).

**Table 2:** Comparison between patients who relapsed and those who achieved remission

	Relapse (n = 24)	Remission (n = 24)	*P*-value
Age (year)	12 (9-14)	11.5 (9.75-14)	.787
Male sex	7 (29.2)	6 (25)	.745
Family history of AITD	11 (45.8)	3 (12.5)	.**024**
BMI_(SDS)_	−0.19 (−1.75-0.19)	−1 (−1.68 to −0.46)	.842
Thyromegaly	23 (96)	22 (92)	.551
Orbitopathy	8 (35)	2 (8)	.**036**
Weight loss	16 (66.7)	11 (45.8)	.146
Tachycardia	20 (83.3)	14 (58.3)	.057
Severe clinical presentation	16 (69.6)	12 (50)	.172
Pubertal stage			.934
T1	9 (38)	9 (37.5)	
T2-T4	7 (29)	6 (25)	
T5	8 (33)	9 (37.5)	
TSH (mIU/L)	0.005 (0.005-0.01)	0.01 (0.005-0.01)	.179
FT4 (pmol/L)	49.29 (43.62-69.62)	49.54 (34.74-67.95)	.992
FT3 (pmol/L)	23.71 (18.66-32.74)	21.49 (13.24-25.39)	.170
FT3/FT4 ratio	0.46 (0.42-0.50)	0.36 (0.32-0.44)	.**001**
TRAb			.422
Negative	0 (0)	1 (4.2)	
<2**×**	0 (0)	1 (4.2)	
2-5**×**	6 (25)	6 (25)	
5-10**×**	5 (20.8)	8 (33.4)	
>10**×**	13 (54.2)	8 (33.4)	
TPOAb			.420
Negative	3 (12.6)	8 (33.4)	
<2**×**	1 (4.2)	2 (8.4)	
2-5**×**	6 (25)	4 (16.6)	
5-10**×**	3 (12.6)	3 (12.6)	
>10**×**	11 (45.8)	7 (29.2)	
TgAb			.456
Negative	6 (25)	8 (33.4)	
<2**×**	4 (16.6)	5 (20.8)	
2-5**×**	2 (8.4)	4 (16.6)	
5-10**×**	1 (4.2)	2 (8.4)	
>10**×**	11 (45.8)	5 (20.8)	
RAPD (mm)	2 (0.5-3)	0 (0-1.5)	.**018**
LAPD (mm)	3 (0-5)	0 (0-2)	.**024**
β blocker	9 (39.2)	11 (47.8)	.552
Time FT3 normalization (days)	60 (30-60)	30 (20-30)	.**038**
Time FT4 normalization (days)	35 (27.5-60)	30 (20-30)	.051
TRAb negativization	20 (85.2)	22 (93.6)	.601
Time to TRAb negativization (months)	24 (18-36)	24 (12-36)	.378
TSH before withdrawal (mcu/mL)	3.06 (1.40-5.88)	3.03 (1.80-4.72)	.179
FT4 before withdrawal (pmol/L)	12.9 (11.58-15.31)	15.44 (11.96-16.73)	.132
FT3 before withdrawal (pmol/L)	5.63 (5.24-6.34)	5.33 (4.83-5.84)	.105
FT3/FT4 ratio before withdrawal	0.43 (0.35-0.48)	0.33 (0.28-0.37)	**<**.**001**
Dose of methimazole before withdrawal (mg/kg/day)	0.06 (0.04-0.08)	0.05 (0.04-0.06)	.056
Duration of first cycle of therapy	2.3 (2-3)	3 (2-3.75)	.196
Follow-up after withdrawal	3.2 (2.3-6.1)	5 (3.4-8.2)	.090

Data are presented as median (interquartile range) or number (percentage).

Abbreviations: AITD, autoimmune thyroid disease; BMI, body mass index; FT3, free T3; FT4, free T4; LAPD, left anteroposterior diameter; RAPD, right anteroposterior diameter; TgAb, thyroglobulin antibodies; TPOAb, thyroperoxidase antibodies; TRAb, thyrotropin receptor autoantibodies.

Ultrasound data were available for 73 patients. About the anteroposterior diameter of the thyroid lobes, a significant difference was identified between the 2 groups; patients who relapsed had a greater anteroposterior diameter of both thyroid lobes than patients who went into remission.

The FT3/FT4 ratio was significantly higher in patients who relapsed both at diagnosis and at the time of ATD withdrawal. Median FT3/FT4 ratio at diagnosis was 0.46 in patients who relapsed compared with 0.36 in those who achieved remission (*P* .001). At ATD withdrawal, the corresponding values were 0.43 and 0.33, respectively (*P* < .001) (Table 2).

**Figure 2:**
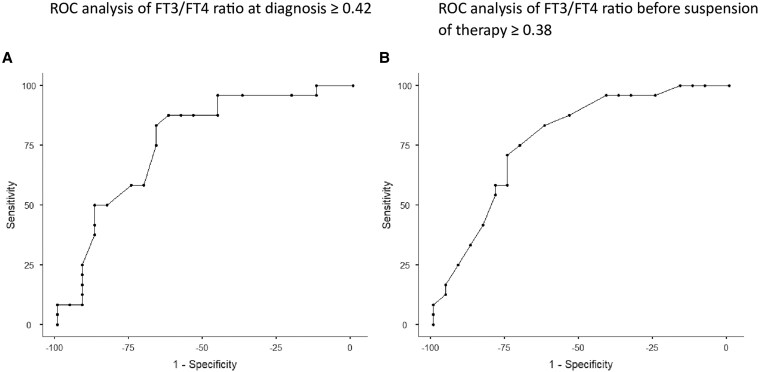
ROC curves evaluating the discriminatory performance of the marker in distinguishing patients who experienced relapse from those who remained in remission at 2 different time points. (A) ROC curve based on marker value at diagnosis. (B) ROC curve based on marker value prior to treatment withdrawal. The AUC was 0.76 (95% CI 0.64-0.91) at diagnosis and 0.78 (95% CI 0.63-0.90) prior to treatment withdrawal, indicating good and consistent discriminatory performance. Abbreviations: AUC, area under the curve; CI, confidence interval; ROC, receiver operating characteristic.

Receiver operating characteristic analysis identified an FT3/FT4 ratio ≥0.42 at diagnosis as the optimal cutoff for predicting relapse [area under the curve (AUC) 0.76]. This cutoff yielded a sensitivity of 83% and a specificity of 66% (Table 3, Fig. 2). An FT3/FT4 ratio ≥0.38 at ATD withdrawal was also associated with increased relapse risk (AUC 0.77; sensitivity 71%; specificity 75%) (Table 3, Fig. 2). We also identified a cutoff of ≥53 days for the normalization time of FT3, which was higher in patients who relapsed (AUC 0.67; sensitivity 54%, specificity 79%) (Table 3).

**Table 3:** Optimal cutoff capable of predicting relapse after ATD withdrawal

Predictor	Specificity (%)	Sensitivity (%)	PPV (%)	NPV (%)	AUC
FT3/FT4 ratio at diagnosis ≥ 0.42	66	83	71	80	0.76
FT3/FT4 ratio before withdrawal ≥ 0.38	75	71	72	74	0.78
Time FT3 normalization ≥ 53 days	79	54	72	63	0.67

Abbreviations: ATD, antithyroid drug; AUC, area under the curve; FT3, free T3; FT4, free T4; NPV, negative predictive value; PPV, positive predictive value.

Finally, a statistical analysis was conducted using logistic regression to identify independent risk factors of relapse for patients who discontinue therapy. The variables described previously for which a significant difference was found were included in this analysis. Table 4 shows the results of the univariate and multivariate analyses. In univariate logistic regression analyses, a family history of autoimmune thyroid disease, orbitopathy, FT3/FT4 ratio at diagnosis ≥0.42, FT3/FT4 ratio before withdrawal ≥0.38, and delayed FT3 normalization (≥53 days) can be considered predictors of relapse (Table 4). In multivariate analysis, only FT3/FT4 ratio ≥0.42 at diagnosis (odds ratio 7.18; *P* .012) and the presence of orbitopathy (odds ratio 5.39, *P* .042) remained independent predictors of relapse.

**Table 4:** Univariate and multivariate logistic regression analysis of factors associated with relapse

Predictor	*P* (univariate)	Odds ratio (univariate)	*P* (multivariate)	Odds ratio (multivariate)
Family history of AITD	**.016**	5.92 (1.386-25.300)	.082	4.880 (0.713-33.461)
Orbitopathy	.**039**	5.87 (1.090-31.560)	.**042**	5.39 (0.672-43.280)
FT3/FT4 ratio at diagnosis ≥ 0.42	**<**.**001**	12.143 (3.030-48.666)	.**012**	7.180 (1.250-41.190)
FT3/FT4 ratio before withdrawal ≥ 0.38	.**003**	7.28 (2.034-26.102)	.067	5.140 (0.890-29.750)
Time FT3 normalization ≥ 53 days	.**020**	4.49 (1.260-16.010)	.817	1.920 (0.340-10.690)

Abbreviations: AITD, autoimmune thyroid disease; FT3, free T3; FT4, free T4.

In support of the importance of the FT3/FT4 ratio, we made 2 comparisons reported in Tables 5 and 6. The first involves patients who went into remission after the first cycle of therapy and those who went into remission after the second cycle. It is evident that the former had a higher ratio both at diagnosis and at the first discontinuation of therapy. The second comparison involves all patients who went into remission and all patients undergoing definitive therapy, with the latter having a significantly higher ratio.

**Table 5:** Comparison of FT3/FT4 ratio at diagnosis and before first suspension between patients who achieved remission after first cycle of therapy and patients who achieved remission after second cycle of therapy

	Remission after I cycle (24)	Remission after second cycle (8)	*P*-value
FT3/FT4 ratio diagnosis (median)	0.360	0.440	.093
FT3/FT4 ratio before first suspension (median)	0.330	0.410	.**034**

Abbreviations: FT3, free T3; FT4, free T4.

**Table 6:** Comparison of FT3/FT4 ratio at diagnosis between all patients in remission and all patients who underwent definitive therapy

	Remission (32)	Definitive therapy (28)	*P*-value
FT3/FT4 ratio diagnosis (median)	0.405	0.450	**.003**

Abbreviations: FT3, free T3; FT4, free T4.

## Discussion

The overall remission rate in pediatric age reported in the literature after 2 years of ATD is only 20% to 30% (4). Duration of medical therapy is 1 of the few modifiable risk factors, with a recent systematic review reporting higher remission rates with duration of medical therapy exceeding 2 to 3 years (3, 4). In our cohort, the remission rate after a median treatment duration of approximately 3 years was 30%, consistent with existing literature and current European Thyroid Association recommendations.

Among the prognostic factors for remission reported in the literature, 1 is certainly represented by age at diagnosis. In particular, a later onset represents a prognostic factor for remission (5). In our study, no significant difference was found regarding age at diagnosis between patients who went into remission and patients who relapsed or who underwent definitive therapy, and no significant differences were found for pubertal stage either. However, comparing patients who have received definitive therapy to those who have not reveals that the former group has a significantly lower average age at diagnosis (9.63 years compared to 11.4 years). An important role is played by positive family history, which in the literature has been shown to be a negative prognostic factor when present (21), as also in our study. Regarding clinical manifestations, the most notable findings were associated with orbitopathy, which, when present, elevates the risk of recurrence after therapy is discontinued. Orbitopathy emerged as an independent predictor of relapse, consistent with previous reports linking extrathyroidal manifestations to a more aggressive autoimmune phenotype (22).

TRAbs are the most extensively studied factor in the literature regarding their prognostic significance for the outcomes of AH. TRAb levels also represent the most extensively studied biochemical marker in GD prognosis. While several studies have associated higher TRAb titers or delayed negativization with relapse (23, 24), others, including our study, failed to demonstrate a predictive role of TRAb levels at diagnosis (25). This discrepancy may reflect assay heterogeneity, differences in timing of measurement, and the dynamic nature of TRAb kinetics rather than a lack of biological relevance (7, 26-28).

Another interesting point concerns the normalization time of FT3 and FT4 values after the start of medical therapy. Several studies involving pediatric patients with AH compared those who went into remission with those who relapsed, focusing on the time to normalization of free thyroid hormones following the initiation of medical therapy (24-28). Only one study found a significant difference in the time to normalization of FT4, which was on average higher in patients who relapsed (25). Our study showed that the time to normalization of FT3 can represent a relevant prognostic factor for disease outcome. In the comparison between patients who went into remission and those who relapsed, a significant difference was observed only in the time to normalization of FT3, which was longer in patients who relapsed. The cutoff ≥ 53 days proved to increase the risk of relapse. The size of the thyroid represents a negative prognostic factor for remission; in fact, patients with a large goiter have a greater risk of recurrence and negative outcome (11, 28). In some studies, the “clinical” dimensions of the thyroid were taken into consideration by measuring the longitudinal diameters; in other studies, the ultrasound dimensions were analyzed.

The most novel and clinically relevant finding of our study is the prognostic value of the FT3/FT4 ratio. Importantly, absolute FT3 and FT4 concentrations at diagnosis were not associated with relapse in our cohort, whereas their ratio showed a robust predictive value. This finding highlights the limitations of relying solely on hormone concentrations to assess disease severity and supports the concept that relative hormone production patterns may better reflect underlying pathophysiology. These data are consistent with that in the literature, which states that distinct patterns of thyroid hormone secretion at diagnosis have a worse outcome, especially in terms of a greater likelihood of relapse after discontinuing therapy (5, 11, 25, 28, 29). The identification of a diagnostic cutoff (FT3/FT4 ≥ 0.42) with good sensitivity and specificity further enhances the potential clinical utility of this parameter. The FT3/FT4 ratio has often been used in studies for the differential diagnosis between GD, Hashimoto's thyroiditis (HT), and subacute thyroiditis, all potentially causes of thyrotoxicosis (30, 31). In fact, these studies demonstrated how patients with GD have a significantly higher FT3/FT4 ratio compared to patients with HT and subacute thyroiditis, who present a value similar to that of healthy controls. The ratio of serum T3 to T4 concentrations has also been recommended as a useful indicator to differentiate patients with GD from those with HT. In GD, T3 concentration will often be higher than T4 due to hyperfunction of the thyroid itself, while in HT the T3/T4 ratio may not be changed due to destruction of the thyroid gland. The excess thyroid hormone could be the result of either increased thyroid T3 production or increased conversion of peripheral T4 to T3, mediated by upregulation of iodothyronine deiodinases, particularly D1 and D2. Experimental and clinical studies have demonstrated that in hyperthyroidism, thyroidal D1 activity may account for up to 50% to 60% of circulating T3, supporting the concept of a “T3-predominant” GD phenotype (17, 18). The high production of thyroid T3 could explain why some patients have persistently high T3 measurements, with normal or low T4 values during treatment, configuring what we described in the introductory section as T3-predominant GD. In a study, D1 and D2 activities were evaluated in thyroid tissue samples from patients with T3-predominant GD treated with methimazole before thyroidectomy (32). Interestingly, not only was an increased activity of both enzymes observed but also a correlation between intrathyroidal D2 activity and the serum ratio between FT3 and FT4. However, it is important to note that the measurement of T3 and T4 is influenced by thyroid hormone-binding proteins, justifying the more frequent use of FT3 and FT4 measurement in clinical practice. In a few studies, the FT3/FT4 ratio has been used as a prognostic factor for the outcome of AH (33-35). Only in one of these, conducted on adult patients, was there a significant difference between patients who went into remission and patients who relapsed after suspension of therapy, with the latter having a higher FT3/FT4 ratio at diagnosis (35). In our study, the FT3/FT4 ratio proved to be an excellent prognostic factor for disease outcome, both at diagnosis and before the suspension of therapy. We point out that the significantly higher FT3/FT4 ratio before withdrawal observed in the relapse group likely reflects the combined effect of relatively higher FT3 concentrations together with relatively lower FT4 concentrations, even when individual hormone differences do not reach statistical significance. This supports the concept that the ratio captures subtle shifts in relative hormone production patterns that may not be evident when analyzing absolute values separately.

The analyses involving patients who underwent definitive therapy should be interpreted with caution. The decision to proceed to surgery or radioiodine was based on clinical judgment and disease course, and the FT3/FT4 ratio was not used as a decision-making parameter in routine practice during the study period. These data were included for descriptive purposes only, to further contextualize disease severity and to indirectly support the hypothesis that the FT3/FT4 ratio may be a useful marker of disease outcome, rather than to imply any causal relationship with treatment selection.

From a clinical perspective, our findings have relevant implications. Incorporation of the FT3/FT4 ratio into routine assessment at diagnosis and before ATD withdrawal may help identify patients at higher risk of relapse, in whom prolonged medical therapy or earlier consideration of definitive treatment may be warranted. Conversely, patients with lower ratios may be suitable candidates for earlier treatment discontinuation, potentially reducing unnecessary drug exposure. This study has several limitations. Its retrospective design and single-center setting may limit generalizability, and the relatively small sample size restricts subgroup analyses. Thyroid ultrasound assessments were not standardized across operators, and thyroid volume could not be calculated in all patients. Nevertheless, the long follow-up duration and the consistency of the association between the FT3/FT4 ratio and relapse across multiple analyses strengthen the validity of our findings.

## Conclusions

In conclusion, current guidelines suggest suspending therapy after at least 3 years to increase the possibility of remission. In our study, the remission rate after 3 years was 30%; these findings, consistent with the literature, indicate that it is appropriate to attempt therapy discontinuation based on clinical history and prognostic factors. The identification of prognostic factors for disease outcome represents a very useful tool for clinicians for optimal management of the follow-up of each patient, thus allowing individualization of the diagnostic-therapeutic path.

The FT3/FT4 ratio is a readily available and biologically meaningful biomarker that independently predicts relapse after ATD withdrawal in pediatric AH. Assessment of this ratio at diagnosis and prior to treatment discontinuation may support individualized decisions regarding treatment duration and timing of withdrawal. Integration of the FT3/FT4 ratio into clinical practice has the potential to improve risk stratification and optimize long-term management in children with GD. Prospective multicenter studies are warranted to validate these findings and to define evidence-based clinical algorithms incorporating this parameter.

## Data Availability

Original data generated and analyzed during this study are included in this published article.

## Abbreviations

AH: autoimmune hyperthyroidism
ATD: antithyroid drugs
AUC: area under the curve
FT3: free T3
FT4: free T4
GD: Graves’ disease
HT: Hashimoto’s thyroiditis
TRAb: thyrotropin receptor autoantibody
